# The exometabolome of *Clostridium thermocellum* reveals overflow metabolism at high cellulose loading

**DOI:** 10.1186/s13068-014-0155-1

**Published:** 2014-10-21

**Authors:** Evert K Holwerda, Philip G Thorne, Daniel G Olson, Daniel Amador-Noguez, Nancy L Engle, Timothy J Tschaplinski, Johannes P van Dijken, Lee R Lynd

**Affiliations:** Thayer School of Engineering, Dartmouth College, Hanover, NH 03755 USA; BioEnergy Science Center, Oak Ridge, TN 37830 USA; Mascoma Corporation, Lebanon, NH 03766 USA; Department of Bacteriology, University of Wisconsin-Madison, Madison, WI 53706 USA; Biosciences Division, Oak Ridge National Laboratory, Oak Ridge, TN 37830 USA; Emeritus Industrial Biotechnology of Delft University of Technology, Delft, BC 2628 The Netherlands

**Keywords:** *Clostridium thermocellum*, Cellulose fermentation, Isobutanol, 2,3-butanediol, Amino acids, High solids, Fusel alcohols

## Abstract

**Background:**

*Clostridium thermocellum* is a model thermophilic organism for the production of biofuels from lignocellulosic substrates. The majority of publications studying the physiology of this organism use substrate concentrations of ≤10 g/L. However, industrially relevant concentrations of substrate start at 100 g/L carbohydrate, which corresponds to approximately 150 g/L solids. To gain insight into the physiology of fermentation of high substrate concentrations, we studied the growth on, and utilization of high concentrations of crystalline cellulose varying from 50 to 100 g/L by *C. thermocellum*.

**Results:**

Using a defined medium, batch cultures of *C. thermocellum* achieved 93% conversion of cellulose (Avicel) initially present at 100 g/L. The maximum rate of substrate utilization increased with increasing substrate loading. During fermentation of 100 g/L cellulose, growth ceased when about half of the substrate had been solubilized. However, fermentation continued in an uncoupled mode until substrate utilization was almost complete. In addition to commonly reported fermentation products, amino acids - predominantly L-valine and L-alanine - were secreted at concentrations up to 7.5 g/L. Uncoupled metabolism was also accompanied by products not documented previously for *C. thermocellum*, including isobutanol, *meso*- and RR/SS-2,3-butanediol and trace amounts of 3-methyl-1-butanol, 2-methyl-1-butanol and 1-propanol. We hypothesize that *C. thermocellum* uses overflow metabolism to balance its metabolism around the pyruvate node in glycolysis.

**Conclusions:**

*C. thermocellum* is able to utilize industrially relevant concentrations of cellulose, up to 93 g/L. We report here one of the highest degrees of crystalline cellulose utilization observed thus far for a pure culture of *C. thermocellum*, the highest maximum substrate utilization rate and the highest amount of isobutanol produced by a wild-type organism.

**Electronic supplementary material:**

The online version of this article (doi:10.1186/s13068-014-0155-1) contains supplementary material, which is available to authorized users.

## Background

*Clostridium thermocellum* is a model organism for the production of cellulosic biofuels in a consolidated bioprocessing (CBP) configuration, because of its plant cell wall-solubilizing enzyme complex, the cellulosome, and its ability to ferment cellodextrins to ethanol [1-3]. Although this organism has been studied for more than 60 years [4], there are still many gaps in our understanding of its physiology. For example, formate production was reported by McBee in 1954 [5], but it took more than 50 years before formate was shown to be a significant fermentation end product [6]. Ellis *et al*. reported in 2012 that *C. thermocellum* produces extracellular amino acids in significant amounts [7]. The concentrations of extracellular amino acids increased when metabolic pathways for lactate and acetate were knocked out [8]. With a combination of gene deletions and enzymatic assays it has been shown that *C. thermocellum* has atypical glycolysis and lacks pyruvate kinase [9]. The recent development and application of genetic tools [10-12] has accelerated both applied and fundamental investigations of this organism.

Most of our current understanding of *C. thermocellum* physiology is based on fermentations with carbohydrate loadings of <10 g/L [7,13-31]. There are, however, a few notable exceptions. Tailliez *et al*. cultured a mutated strain of *C. thermocellum* that utilized 63 g/L out of 70 g/L cellulose (MN300) and produced 14.5 g/L ethanol [32]. Wang *et al.* used a fed-batch cellulose fermentation with a maximum utilization of 35 g/L cellulose (Solka-Floc®) to test mutant strains of *C. thermocellum*. Their study also included a co-culture of *C. thermocellum* with *Thermoanaerobacterium thermosaccharolyticum* that grew on 80 g/L solids (Solka-Floc) with three quarters of the substrate utilized and a 100 g/L cornstover co-culture with 37% of the substrate utilized [33]. Hogsett reported a set of sequential high solids fermentations with initial cellulose loadings of 26, 47, and 72 g/L and a maximum cellulose utilization of 65 g/L [34].

*Clostridium thermocellum* strains are routinely isolated from saccharolytic microbial communities [4,35,36], and some lab strains of *C. thermocellum* have turned out to be stable co-cultures of cellulolytic and non-cellulolytic species [37-39]. It has been hypothesized that this stability may be due to cross-feeding of vitamins and other compounds between strains [40,41], with positive effects on the fermentation of cellulose [42,43]. The beneficial effects of co-culturing were recently also demonstrated by Argyros *et al.*, who showed that a co-culture of engineered strains of *C. thermocellum* and *T. saccharolyticum* was able to ferment 83 g/L of cellulose into an impressive 38 g/L ethanol (80% of the theoretical maximum), while a pure culture of the same *C. thermocellum* strain produced only 14 g/L of ethanol when cultured on 40 g/L cellulose [10]. Application in industrial processes is likely to require carbohydrate concentrations of 100 g/L carbohydrate or more [44], corresponding to lignocellulose loadings of at least 150 g/L on a dry matter basis.

This work was undertaken to study the physiology of *C. thermocellum* at high cellulose loading to guide further metabolic engineering aimed at improvement of ethanol production.

## Results and discussion

### Fermentation of increasing cellulose loadings

**Figure 1:**
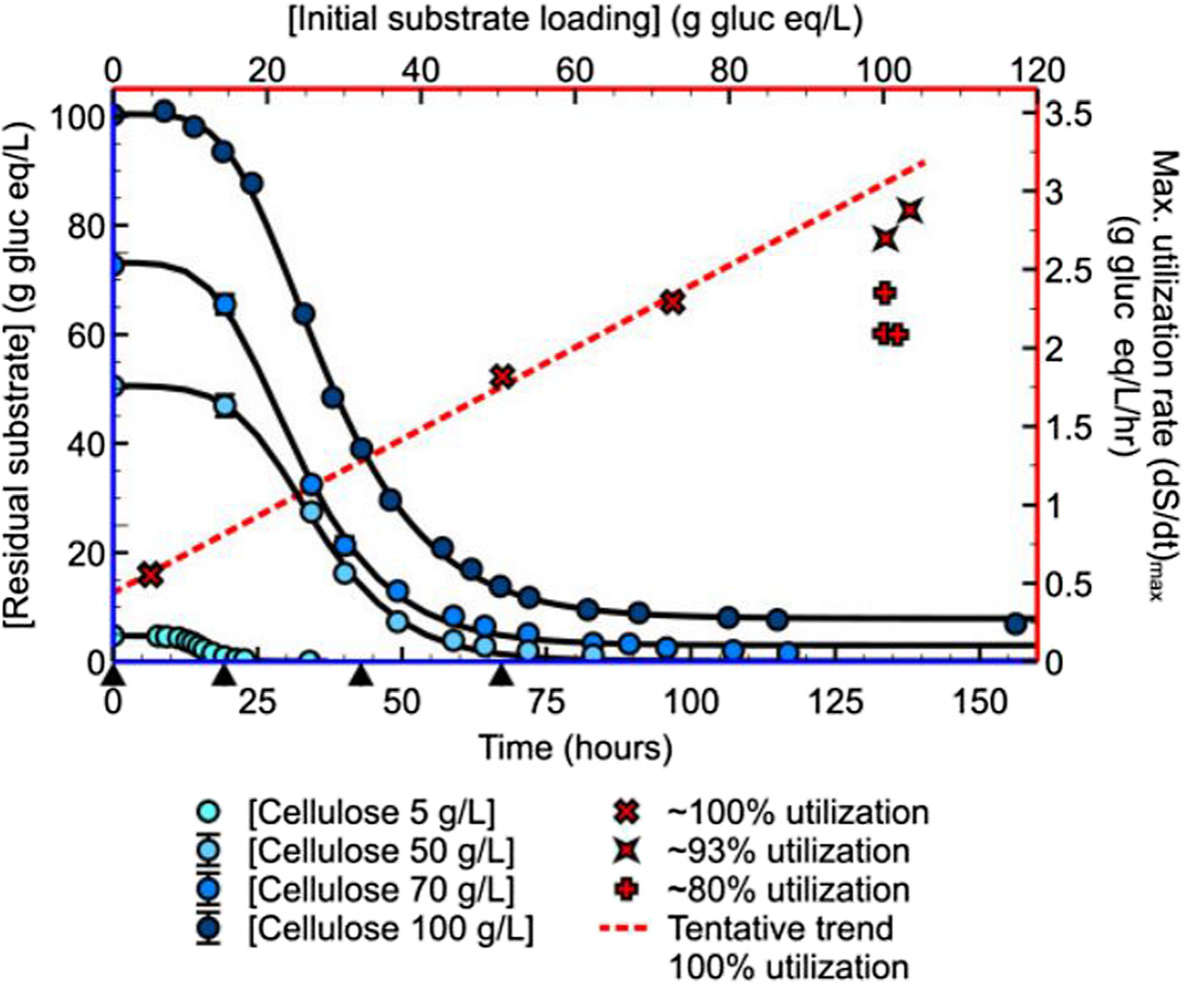
**Residual substrate concentrations for 5, 50, 70, and 100 g/L cellulose in gram glucose equivalents/L, and maximum substrate utilization rates versus initial substrate loading.** Black triangles on the bottom *x*-axis indicate time points where additional vitamins were added for the 100 g/L fermentation. An additional data set of a 5 g/L cellulose fermentation is added. For accurate substrate values shown here, see Table 1.

Three different substrate loadings (50, 70 and 100 g/L) of crystalline cellulose (Avicel PH105) were tested under the same fermentation conditions and nutrient concentrations (Figure 1). Out of the three substrate loadings only the 100 g/L loading did not reach full conversion of the substrate (not shown in Figure 1). Repeated 100 g/L fermentations (n = 3) showed that the conversion was stable around 80% of 100 g/L (see Table 1). Addition of nitrogen to the growth medium in the form of ammonium sulfate (5 mL*200 g/L (NH_4_)_2_SO_4_), ammonium hydroxide (14.8 N NH_4_OH) as base addition for pH control, and urea (8 mL*500 g/L (NH_2_)_2_CO) did not increase conversion. Cysteine, added as a reducing agent to the medium, can be a possible secondary source of nitrogen and sulfur to cells. Measuring nitrogen in the form of urea or ammonia (NH_3_) via enzymatic assay showed the presence of residual nitrogen in the medium, which confirmed that the lack of complete conversion of the substrate was not caused by nitrogen limitation.

**Table 1:** **Initial and final substrate concentrations and rate data for all fermentations shown in Figure** 1

**Initial [substrate] (g gluc eq/L)**	**Final [substrate] (g gluc eq/L)**	**Utilized (g gluc eq/L) (% utilization)**	**Maximum substrate utilization rate (g gluc eq/L/hr)**
4.8	0.04	4.76 (99)	0.56
50.6	0	50.6 (100)	1.82
72.7	0.72	71.08 (99)	2.29
100.2	19.2	81 (81)	2.35
100.1	22.6	77.5 (77)	2.09
101.8	19.1	82.7 (81)	2.09
100.3	6.9	93.4 (93)	2.70
103.4	6.7	96.7 (94)	2.88

Actual concentrations of substrate are shown in gram glucose equivalents/L (g gluc eq/L) since Avicel PH105 contains xylose and insoluble residual content, and has a moisture content of around 5%. The three categories of maximum substrate utilization rates shown in Figure 1 are based on percentage utilization as shown here (approximately 80% utilization, 93% utilization and 100% utilization).

For various time points, the supernatant of the fermentation broth was analyzed for trace metals/elements B, Mn^2+^, Co^2+^, Ni^2+^, Cu^2+^, Zn^2+^, Mo^2+^, Na^+^, Fe^2+^, Mg^2+^, and Ca^2+^ and the vitamins pyridoxamine-2*HCL, cyanocobalamin (B_12_) and para-aminobenzoic acid (PABA). Biotin was not assayed (see Methods). Trace elements were detected throughout the duration of the fermentation, although Cu^2+^ and Mn^2+^ decreased in concentration during the fermentation. Doubling the trace elements solution did not result in an increase in substrate utilization. Vitamin B_12_ was not detected in the fermentation broth by the vitamin B panel analysis, but was detected at the correct concentration in the stock solution. The pyridoxamine-2*HCl concentration decreased to zero at 50% conversion, and niacinamide (vitamin B_3_) was detected halfway through the fermentation. It was not clear how the analysis failed to detect vitamin B_12_ in the medium during the fermentation process. To avoid pyridoxamine depletion and to control for the effect of possible spontaneous degradation of vitamin B_12_ under fermentation conditions, fresh vitamin solution was added at several times during the fermentation, rather than all at once in the beginning. By adding supplemental vitamins at four different times (Figure 1) during the fermentation, substrate utilization increased to 93%. Vitamin analysis showed pyridoxamine present at all time points, but neither vitamin B_12_ nor B_3_ was detectable.

*C. thermocellum* achieved complete conversion at substrate loadings up to 70 g/L and initially achieved 80% conversion at substrate loadings of 100 g/L. The addition of extra aliquots of vitamins alleviated restrictions, and utilization increased from 80% to 93% for 100 g/L substrate loadings. Whether this was due solely to depleted pyridoxamine, or whether other vitamins also played a role would require a more detailed analysis. The standard concentration of vitamins used for the experiments discussed here is according to Hogsett [34] and is ten times higher than that described by Johnson *et al.* [15]. The highest amount of utilized cellulose we observed was 96.7 g/L out of 103.4 g/L (about 94%, see Table 1), one of the highest values reported for a pure culture of *C. thermocellum* thus far [45].

### Rates of cellulose utilization

The fermentation data for all three high cellulose loadings and the 5 g/L fermentation in Figure 1 have been fitted with a sigmoidal curve [29], from which a maximum rate was determined and plotted versus the initial substrate loading (Figure 1). The increase of the utilization rate with the increase in substrate loading of 5 to 70 g/L fermentations (these fermentations reached about 100% conversion, see Table 1) is fitted with a straight line. Data points for five 100 g/L initial cellulose loadings are also shown; three fermentations reached 80% conversion and two reached 93% conversion (see Table 1). The maximum utilization rates for the 80% conversion fermentations are lower than those for the 93% conversion fermentations, although all fermentations started with the same substrate loadings (corresponding to equal available substrate surface area). An increase in utilization because of increased vitamins also resulted in an increased maximum utilization rate, and the utilization rates of the two approximately 93% fermentations are close to the fitted curve, based on the data for lower initial substrate concentration at which 100% conversion was observed.

The time it takes to utilize 50 g/L or 93 g/L is very similar with the same size inoculum (0.5%), as is shown by the increasing maximum rate of substrate utilization with increasing solids loading (except for the 100 g/L: 80% conversion, non-optimized fermentations, where the rate is equal to or lower than that of the 70 g/L substrate loading). The maximum substrate utilization rate at 100 g/L loading (about 2.9 g/L/h) is among the highest reported for a pure culture of *C. thermocellum* (2.5 g/L/h, [34]).

### Carbon balance for commonly reported end products

**Figure 2:**
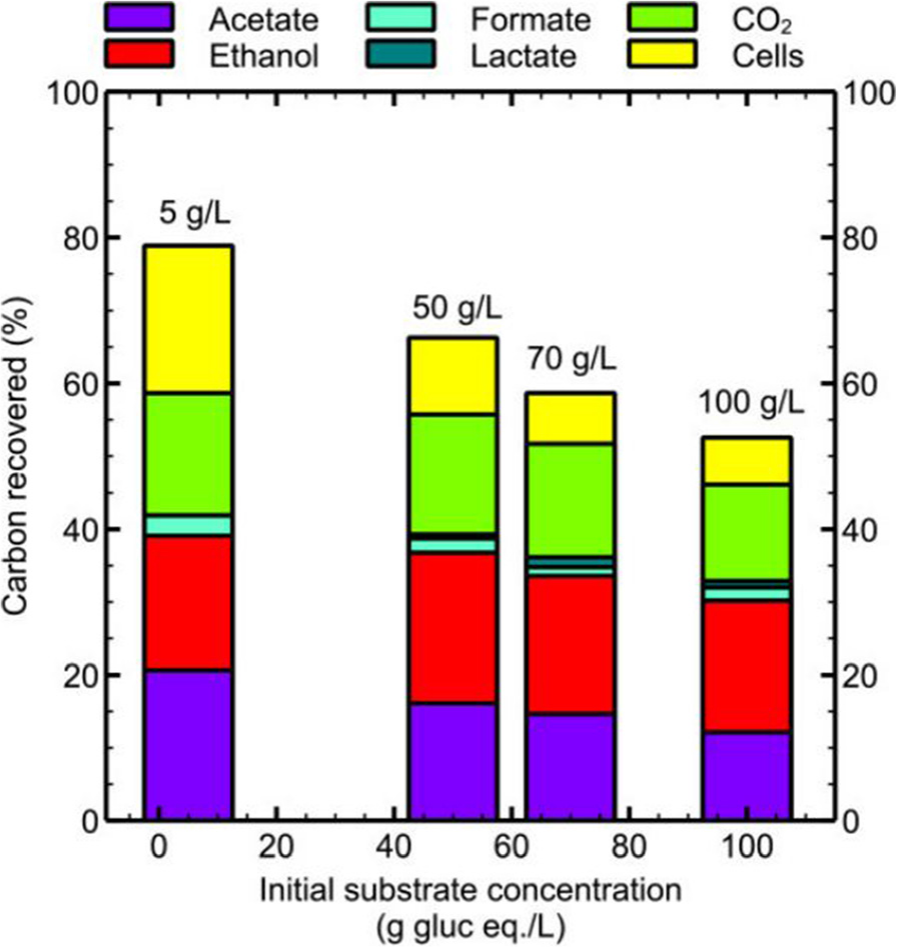
**Carbon balance over commonly reported fermentation end products.** The fermentation end products acetate, ethanol, formate, lactate, CO_2_ and cells as percentage of carbon of the initial substrate loading. All fermentations reached about 100% conversion, except the 100 g/L substrate loading, which reached 93% conversion. All balances are based on the difference between start and endpoint samples.

**Figure 3:**
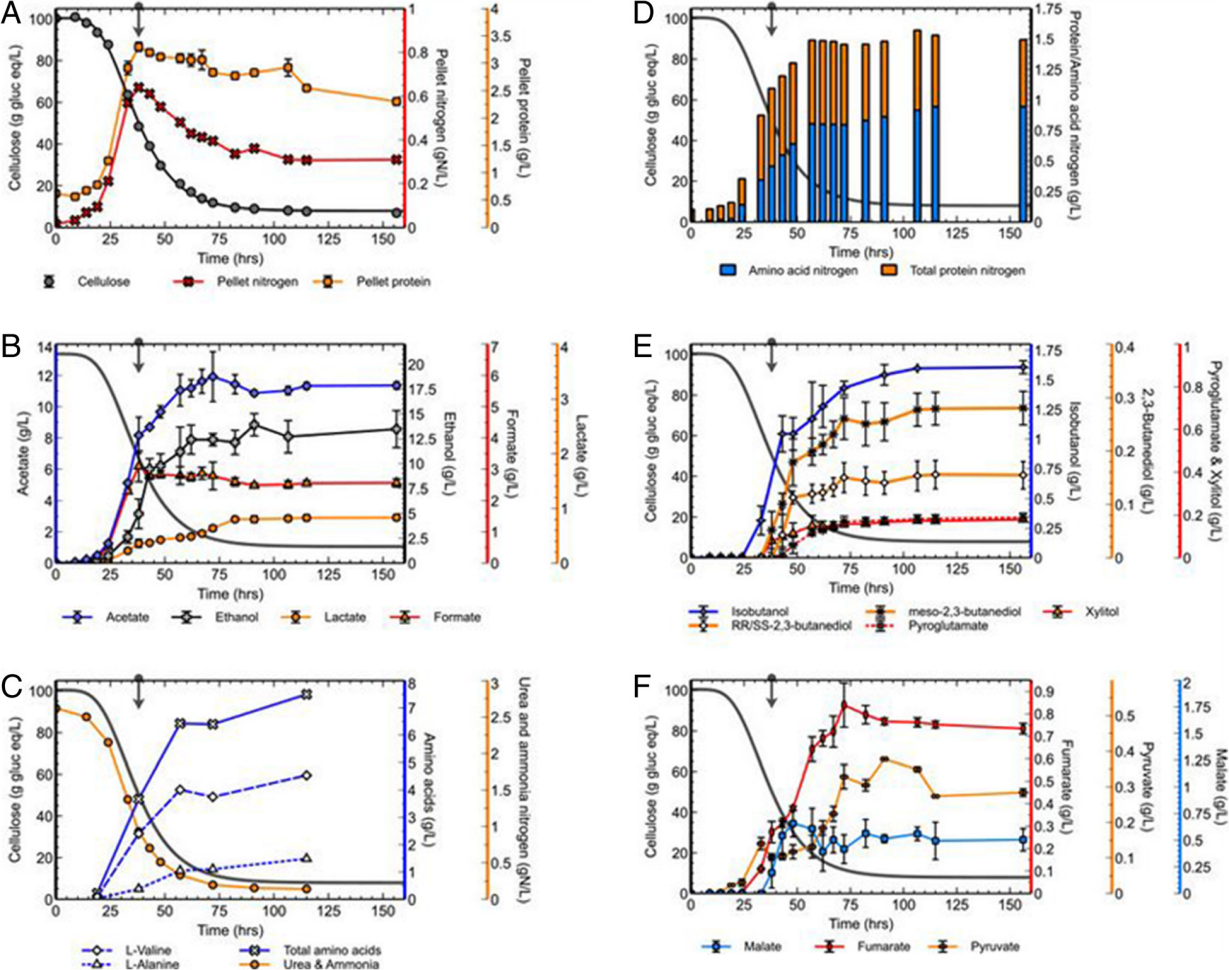
**Residual substrate and products concentration during fermentation.** Residual substrate, pellet protein, and pellet nitrogen concentrations **(A)**; acetate, ethanol, formate and lactate concentrations **(B)**; residual medium nitrogen and amino acid concentrations **(C)**; amino acid nitrogen and total (supernatant and pellet) protein nitrogen concentrations **(D)**; isobutanol, *meso*-2,3-butanediol, RR/SS-2,3,-butanediol, pyroglutamate, and xylitol concentrations **(E)**; pyruvate, malate and fumarate concentrations **(F)**. Figures B-F show the residual substrate concentration curve and an arrow indicating the time point at which cell growth stops in dark gray.

Carbon balances based on the commonly reported fermentation products acetate, ethanol, formate, lactate, cells and carbon dioxide were made for all four different substrate loadings and are shown in Figure 2 (more detailed time courses for the fermentation end products acetate, ethanol, lactate and formate of *C. thermocellum* for the optimized 100 g/L fermentation are shown in Figure 3B). With increasing substrate loading the relative amount of carbon accounted for in these end products decreased from 79% to 53%.

### Cells, residual substrate nitrogen, amino acids and protein

Both pellet protein and pellet nitrogen, considered to be proxies for cell concentration, were measured for the optimized 100 g/L cellulose fermentation with multiple additions of vitamins. At around 50% substrate conversion, the cells stop growing as indicated by the arrows in Figure 3. The remainder of the substrate was nevertheless utilized. At 50% conversion, approximately one-third of the initial medium nitrogen was still present (Figure 3C), and after cell growth stopped the culture utilized a significant amount of additional medium nitrogen. The amount of nitrogen present in cell biomass did not account for all the nitrogen utilized. During and after the cell growth phase, there is a non-trivial amount of nitrogen incorporated into free amino acids; 7.5 g/L of amino acids in total for the fermentation of 93 g/L cellulose utilized, with L-valine and L-alanine predominant at 4.5 g/L and 1.5 g/L respectively (see Figure 3C). The amount of supernatant and pellet protein nitrogen is plotted together with the amount of amino acid nitrogen in Figure 3D. It was determined that about 60% of nitrogen that is being taken up by cells is incorporated into extracellular amino acids.

**Figure 4:**
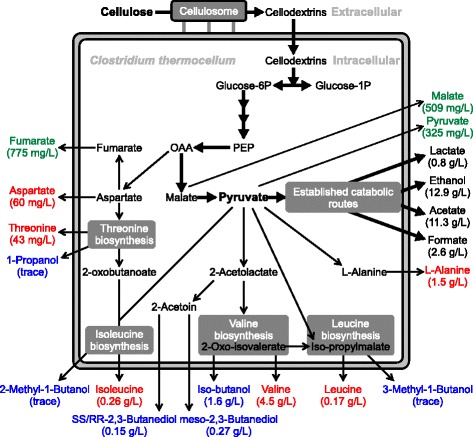
**Fermentation end products and concentrations in an abridged pathway diagram.** Commonly reported compounds (black), amino acids (red), metabolic intermediates (green) and higher alcohols (blue). Bold arrows represent the main catabolic route.

The presence of amino acids in *C. thermocellum* fermentation broth has been reported before, although at lower concentrations and at lower substrate loadings [7,8,46]. Pyruvate serves as a precursor for both L-valine (4.5 g/L) and L-alanine (1.5 g/L) [8] (see Figure 4), the two amino acids produced in the highest concentrations. Other amino acids were produced in significantly lower concentrations; L-glutamate (0.62 g/L), L-isoleucine (0.24 g/L), L-leucine (0.17 g/L), all other measured amino acids did not exceed 100 mg/L. Secreting amino acids is common for rumen bacteria, with L-alanine and L-valine among the most prevalent [47]. L-alanine is a major end product for *Pyrococcus furiosus* during growth on various carbohydrates [48]. A number of other Clostridia species excrete amino acids during the growth phase [49], and Gram-positive bacteria have ten to twenty times higher intracellular amino acids pools than Gram-negative bacteria [50]. It is noteworthy that *C. thermocellum* also contains high amounts of branched-chain fatty acids, which indicates a link for branched-chain fatty acids and branched-chain amino acids L-valine, L-leucine and L-isoleucine [51].

Cessation of growth and decrease in cell concentration for *C. thermocellum* have been observed previously at much lower substrate concentrations and much closer to full substrate conversion than seen here [33,46]. This decrease was attributed to starvation due to decreased access to substrate (reduced accessible surface area) [29]. The decrease in biomass concentration observed here after cessation of cell growth was more rapid for pellet nitrogen than for pellet protein (see Methods and Figure 3A), for which there is no clear explanation.

While the cessation of cell growth due to limitation by nitrogen, vitamins or trace elements is unlikely, limitation of other essential nutrients cannot be ruled out. Accumulation of inhibitory end products and the creation of an ion imbalance can cause cell growth and fermentation to stop [52,53]. However, in our experiments, fermentation was not affected, while biomass ceased to increase. Cell-cell interactions like the quorum sensing mechanism can also impact cell growth. Steiner *et al.* recently found a set of quorum sensing genes in *C. acetobutylicum* [54]. Burell described a species-specific quorum sensing mechanism for a *C. thermocellum*-like isolate from landfill biomass leachate [55], and Wilson *et al.* reported on a gene in *C. thermocellum* with putative peptide quorum sensing function [56].

### Additional fermentation products

During the analysis of fermentation products it became evident that there were more compounds present than there are traditionally attributed to the metabolism of *C. thermocellum*. Identification of these compounds was undertaken, initially based on matching peak residence times to known compounds using gas and liquid chromatography with conformation based on mass spectroscopy (see Additional files 1: Figure S1A, 2: Figure S1B, 3: Figure S2A, 4: Figure S2B, 5: Figure S2C, 6: Figure S2D). Isobutanol and 2,3-butanediols appeared before cell growth stopped (Figure 3E); however, their occurrence did not coincide. Other compounds detected in significant amounts included pyroglutamate, xylitol (Figure 3E), pyruvate, fumarate and malate (Figure 3F). Trace amounts of 1,2,3-butanetriol, 2-methyl-1-butanol (2M1B), 3-methyl-1-butanol (3M1B), and 1-propanol were also detected in fermentation end-samples. Further analysis revealed that both the chiral forms of 2,3-butanediol (SS-/RR-) as well as *meso*-2,3-butanediol were present. Small quantities of xylose (present in low amounts in Avicel PH105) and glucose (as a monomer and in cellodextrins) accumulated towards the end of the fermentation.

Xylitol as a product of (ligno)cellulosic fermentation has been reported for yeast [57,58]. However, it is unclear whether the xylitol found here has strict bacterial origins, or is the result of the abiotic breakdown process of the substrate and interactions with fermentation broth constituents. Pyroglutamate, also known as pyrrolidone carboxylic acid, can be the product of spontaneous degradation reactions of glutamine and of glutamic acid that accelerate at higher temperatures [59,60]. It should be noted that pyroglutamate could have inhibitory effects on growth at high temperatures [61].

Isobutanol biosynthesis branches off from the L-valine biosynthesis pathway in similar ways, 1-propanol and 2-methyl-1-butanol branch off from the threonine/isoleucine biosynthesis pathway, and 3-methyl-1-butanol from the leucine pathway (see Figure 4 and [www.genome.jp/kegg/]) [62,63]. The alcohols formed in this manner are called fusel alcohols, and the pathway in yeast is known as the Ehrlich pathway. Production of fusel alcohols has not been observed previously for *C. thermocellum*, and no specific genes for isobutanol or fusel-alcohol pathways are annotated in the *C. thermocellum* genome [64].

While 1-propanol, 1,2,3,-butanetriol, 2M1B and 3M1B were detected in trace amounts, isobutanol and the butanediols were found in noticeable quantities. There are two ways for isobutanol to be formed from 2-oxo-isovalerate/2-keto-isovalerate from the L-valine biosynthesis pathway (Figure 4): either by a keto-acid decarboxylation reaction into isobutyraldehyde followed by a reduction to isobutanol, or via a keto-acid dehydrogenase reaction into isobutyryl-CoA via a dehydrogenase reaction into isobutylaldehyde and finally by an alcohol dehydrogenase into isobutanol. The only other cellulolytic anaerobe reported to be capable of producing isobutanol is an engineered strain of *C. cellulolyticum* with a maximum concentration of 660 mg/L isobutanol based on a solids loading of 10 g/L cellulose [63].

The appearance of 2,3-butanediol, although in small quantities, is interesting as this would be the first and only example of a thermophilic, cellulolytic microorganism producing 2,3-butanediol [65]. Both the chiral form (RR-/SS-) as well as the *meso*- form of 2,3-butanediol were found, and it is common for wild-type organisms to produce both the *meso* and one of the two chiral molecules [66]. The pathway for the biosynthesis of 2,3-butanediols branches off from the central metabolism via 2-acetolactate at the pyruvate node (see Figure 4). *C. thermocellum* has no annotation of any activity involved in the 2,3-butandiol pathway (as part of the butanoate pathway) from 2-acetolactate onwards. Pathways that include *meso*-2,3-butanediol have been discussed by Siemerink *et al.* [67]. *C. thermocellum* has been used in producing 2,3-butanediol from lignocellulosic substrate but this was in a co-culture in which *Klebsiella pneumoniae* was responsible for generating butanediol [68].

The appearance of these metabolites questioned the purity of the culture. However, 16S rRNA testing, as described in the Methods section, did not result in any irregularities. The 93% conversion-vitamin complemented fermentation discussed here proved to be pure from inoculum throughout the fermentation until the final sample. Further studies are required to establish the nature of the enzymes by which these non-common products are formed and to what extent this is affected by medium components such as citrate and cysteine.

### Overflow metabolism around the pyruvate node

It is not uncommon to find pyruvate in *C. thermocellum* fermentations, even at lower concentrations of substrate [7,10,11]. The concentration of pyruvate found here is relatively low (325 mg/L, see Figure 3F and Figure 4). The additional presence of the glycolysis/citric acid cycle intermediates malate (509 mg/L) and fumarate (775 mg/L) indicates metabolic imbalance (Figure 3F) [9,69]. Desvaux *et al.* reported that *C. cellulolyticum* stopped growing in a defined medium before complete utilization of substrate when cultured on concentrations higher than 6.7 g/L cellulose. Pyruvate accumulation was reported as well [70]. According to their hypothesis, this was caused by the rate of cellulose catabolism exceeding that of pyruvate consumption, leading to an accumulation of intracellular inhibitory compounds. Guedon *et al.* were able to resolve the metabolism and pyruvate overflow of *C. cellulolyticum* by introducing the *Zymomonas mobilis* genes for pyruvate decarboxylase and alcohol dehydrogenase II, resulting an increase in biomass growth and a changed fermentation pattern [71].

All the previously known and newly identified fermentation end products depart from central metabolism at, or downstream of, the pyruvate node, as shown in Figure 4. Around the time that cell growth stops, the carbon flux towards commonly reported fermentation end products decreased (Figure 3B) and flux towards other products increased (Figures 3B and 3F). The appearance of malate, fumarate, pyruvate and isobutanol happens prior to the cessation in cell growth (Figure 3A,E,F). The 2,3-butanediols, pyroglutamate and xylitol appear around the time cell growth stops. An apparent bottleneck exists around the pyruvate node in *C. thermocellum* metabolism, and amino acid and C_4_-solvent production could be a strategy to channel away catabolic intermediates in an attempt to maintain a balanced metabolism. Conclusive proof of this hypothesis will require further experimentation.

We speculate that the very high production of amino acids by *C. thermocellum* reported here is unlikely to occur in nature, but rather is a result of the organism being cultivated under conditions different from those encountered in nature. Among such differences, the absence of other microbial species and the very high substrate and nutrient concentrations seem particularly notable to us. Consumption of carbohydrate fermentation products, in particular H_2_ consumption by methanogens, is known to have large effects on the metabolism of *C. thermocellum* and other saccharolytic microbes [10,13,42,72,73]. In the absence of such consumption and in the presence of very high carbon source concentrations, products accumulate to levels not seen in natural environments, and this accumulation may well trigger metabolic responses. Interspecies cross-feeding is an important source of vitamins in natural environments [40,41,43]. The requirement of *C. thermocellum* for vitamins in pure culture, previously reported [15,34] and also observed here, is likely an indication of this organism existing in nature in close association with other microbes.

Future work co-culturing *C. thermocellum* with other organisms, either a saccharolytic species or a methanogen, would be of interest. Amino acids were not reported by Argyros *et al.* [10], or in any other co-culture study to our knowledge. It appears that the electron and carbon recovery for co-cultures with saccharolytic thermophiles and *C. thermocellum* is higher than that for pure *C. thermocellum* cultures [33].

After accounting for all measured components, the carbon balances for 50, 70 and 100 g/L cellulose loadings increased from 66.3%, 58.7% and 52.5% (Figure 2) to 96.1%, 78.1% and 80.8%, respectively, showing that for the higher concentrations of cellulose still other metabolites remain to be discovered.

## Conclusions

*Clostridium thermocellum* is able to utilize up to 96.7 g/L out of 103.4 g/L cellulose substrate initially present in a defined medium and under controlled conditions. The maximum rate of substrate utilization increases with increasing substrate loading up to 2.9 g glucose/L/h, and is one of the highest rates reported. A concentration of 100 g/L cellulose is equivalent to about 150 g/L of lignocellulosic material based on carbohydrate content, and this result shows that unmodified *C. thermocellum* is able to ferment industrially relevant carbohydrate loadings on a defined medium.

The presence of higher alcohols as fermentation end products is indicative of previously unrecognized metabolic diversity. Further investigation is needed to determine which genes are involved in formation of these overflow metabolites. It would be relevant in this respect to grow *C. thermocellum* with a suitable co-culture partner strain on high cellulose loadings to see whether overflow metabolism occurs and whether cross-feeding of vitamins can eliminate the need for supplemental addition of vitamins during the course of the fermentation.

To our knowledge, this is the first time that SS/RR-/*meso*-2,3-butanediol and isobutanol have been reported as products of the metabolism of *Clostridium thermocellum* or any cellulolytic, thermophilic wild-type microbe cultured on cellulose. The isobutanol concentration of 1.6 g/L is the highest isobutanol concentration observed for a wild-type organism.

## Methods

### Organism and culture purity

*Clostridium thermocellum* DSM 1313 was grown on 5 g/L cellulose in Medium for Thermophilic Clostridia (MTC) [34,46] and stored in 5-mL aliquots at -80°C. The purity of the culture was tested using commercially available 16S rRNA primers from Integrated DNA Technologies (IDT, Coralville IA) (forward primer: AGA GTT TGA TCC TGG CTC AG, reverse primer: ACG GCT ACC TTG TTA CGA CTT). PCR products were sequenced (GENEWIZ Inc.) and compared to the genomes of *C. thermocellum* DSM 1313 and ATCC 27405. No contamination was detected before, during, or at the end of the fermentations described here.

### Bioreactor cultivation

MTC without MOPS buffer was prepared as described previously [46] and contained 5 g/L urea as the nitrogen source. Fermentations were carried out in 2.5- and 1.5-L (1-L working volume) Sartorius Biostat A-plus Sartorius Stedim (Sartorius Stedim, Bohemia, NY) bioreactors with the temperature maintained at 60°C and stirred at 300 rpm. The pH was controlled at 7.0 with a Mettler-Toledo pH probe (Columbus, OH) by the addition of 14.8 N KOH. Base addition, pH and temperature were recorded by the bioreactor setup described in [46]. For vitamin supplementation, aliquots of a 50-fold concentrated vitamin-solution were added at designated time points.

Cellulose suspensions (Avicel PH105, FMC Biopolymer, Philadelphia PA) of 50, 70 and 100 g/L final concentration were autoclaved twice for 3 h. Between autoclaving sessions, the suspensions were stirred and purged with N_2_ gas. The medium was prepared in a 5-L carboy by separately adding each component to the autoclaved cellulose slurry by filter sterilization under anaerobic conditions. The complete medium was then added to the bioreactor under sterile conditions. The bioreactor was inoculated with 5 mL -80°C freezer stock culture grown on 5 g/L Avicel PH105 in MTC (0.5% v/v). The headspace of the bioreactor setup was flushed with N_2_ gas prior to inoculation.

### Pellet nitrogen and protein analysis

Cell biomass expressed as pellet nitrogen was measured by elemental analysis as described in Holwerda *et al.* [29].

1-mL fermentation samples were analyzed for protein content by dividing the sample into a 600-μL supernatant fraction and a 400-μL pellet fraction. The supernatant protein was analyzed by Bradford assay (Bio-Rad protein assay, Bio-Rad, Hercules, CA) against a serial dilution of bovine serum albumin as standard (Thermo Scientific, Rockford, IL). The pellet was rinsed with 1 mL of distilled water, lysed by adding 1 mL of 0.2 M NaOH with 1% w/v SDS, and incubated for 45 min at room temperature with intermittent vortex mixing. After incubation the pH was neutralized by adding 50 μL of 4 N HCl and the mixture was centrifuged at 21,000 rcf (15,000 rpm) for 5 min. The protein in the supernatant of the pellet protein fraction was quantified by bicinchoninic acid (BCA) assay (Pierce Protein assay kit, Thermo Scientific, Rockford IL) with a serial dilution of bovine serum albumin as standard. No distinction was made for cellulosome-associated protein. The amount of nitrogen in protein was taken as 0.161 g/g [74].

### Residual substrate

Residual substrate was determined by quantitative saccharification on triplicate 1-mL samples; see also Holwerda *et al.* [46].

### Fermentation products

Ethanol, lactate, formate, acetate, pyruvate, fumarate, pyroglutamate, isobutanol, *meso*-2,3-butanediol, and RR/SS-2,3-butanediol and xylitol were quantified by HPLC (Waters, Milford, MA) with refractive index (RI) and UV detection using an Aminex HPX-87H column (Bio-Rad, Hercules, CA) with a 5-mM sulfuric acid solution eluent. Malate was quantified by colorimetric assay (BioVision, Milpitas, CA).

Since measurements of CO_2_ are frequently unreliable due to the accumulation of CO_2_ in the liquid fraction, for the purposes of carbon balances CO_2_ production in moles was estimated by stoichiometry using the following formulas:

1 $$ {\mathrm{CO}}_2 = \mathrm{acetate} + \mathrm{ethanol}\ \hbox{--}\ \mathrm{formate} $$

2 $$ {\mathrm{CO}}_2 = \mathrm{acetate} + \mathrm{ethanol}\ \hbox{-}\ \mathrm{formate} + \mathrm{L}\hbox{-} \mathrm{valine} + 2*\mathrm{isobutanol}\ \hbox{--}\ \mathrm{malate} $$

Formula 1 is used when looking at “commonly reported” fermentation products. Formula 2 is a more comprehensive version of formula 1.

To account for solubilized, non-utilized oligosaccharides present at the end of the fermentation, the sugar content of the supernatant was determined by acid hydrolysis and measured by HPLC.

The higher alcohols 1-propanol, 2-methyl-1-butanol, 3-methyl-1-butanol, isobutanol and the 2,3-butanediols were identified by gas chromatography with flame ionization detector (GC/FID)(Agilent 7890A, RESTEK-FAMEWAX, 30 m column, helium carrier) and by matching retention times to standards on two different liquid chromatography columns: H-column (Agilent 1100, Aminex HPX-87H column, Bio-Rad, Hercules, CA, 5 mM H_2_SO_4_, 0.7 mL/min) and P-column (Thermo Spectra, Aminex HPX-87P column, Bio-Rad, Hercules, CA, distilled water, 0.65 mL/min). Refractive index (RI) was used to detect compounds during HPLC analysis. LC-MS was used to identify fumaric acid and pyroglutamate (L-5-oxo-proline) (Thermo LTQ, Aminex HPX-87H column with 0.1% formic acid eluent, 0.7 mL/min). The chiral isomers SS/RR 2,3-butanediol could not be resolved on either the GC or LC.

### Mass spectrometry analysis

Aliquots of supernatants (50 μL) of *Clostridium thermocellum* cultures and sorbitol (aqueous internal standard added to yield 20 ng per μL injected) were transferred by pipette to a vial and stored at -20°C until analyzed. The samples were thawed and concentrated to dryness under a stream of N_2_. The internal standard was added to correct for subsequent differences in derivatization efficiency and changes in sample volume during heating. Dried extracts were dissolved in 250 μL of silylation-grade acetonitrile followed by the addition of 250 μL N-methyl-N-(trimethylsilyl)trifluoroacetamide (MSTFA) with 1% trimethylchlorosilane (TMCS) (Thermo Scientific, Bellefonte, PA), and the samples were then heated for 1 h at 70°C to generate trimethylsilyl (TMS) derivatives. After 1 day, 1-μL aliquots were injected into an Agilent Technologies Inc. (Santa Clara, CA) 5975C inert XL gas chromatograph-mass spectrometer, fitted with an Rtx-5MS with Integra-guard (5% diphenyl/95% dimethyl polysiloxane) 30 m × 250 μm × 0.25 μm film thickness capillary column. The standard quadrupole GC-MS was operated in the electron impact (70 eV) ionization mode, targeting six full-spectrum (50 to 650 Da) scans per second. The gas (helium) flow was 1.3 mL per minute with the injection port configured in the splitless mode. The injection port, MS source, and MS quad temperatures were 250°C, 230°C, and 150°C, respectively. The initial oven temperature was held at 50°C for 2 min and was programmed to increase at 20°C per min to 325°C and held for another 11 min, before cycling back to the initial conditions. A large user-created database (>1,900 spectra) of mass spectral electron ionization (EI) fragmentation patterns of TMS-derivatized compounds as well as the Wiley Registry 8th Edition combined with NIST 05 mass spectral database were used to identify the metabolites of interest to be quantified. The analyses targeted metabolites related to the isobutanol production pathway. Additional analyses of commercial standards confirmed the presence of xylitol, 2-*oxo*-isovalerate, RR/SS-2,3-butanediol, *meso*-2,3-butanediol, and 1,2,3-butanetriol. Peaks were reintegrated and reanalyzed using a key selected ion, characteristic m/z fragment, rather than the total ion chromatogram, to minimize integrating co-eluting metabolites. The extracted peaks of known metabolites were scaled back up to the total ion current using predetermined scaling factors. The scaling factor for the internal standard (sorbitol) was used for unidentified metabolites. Peaks were quantified by area integration and the concentrations were normalized to the quantity of the internal standard recovered, and volume of sample processed, derivatized, and injected [75].

### Amino acids, vitamins and trace elements

Supernatants were analyzed for amino acids (ninhydrin method) and B-vitamins, (“vitamin B-panel” by HPLC) by Aminoacids.com (St. Paul, MN). Para-aminobenzoic acid (PABA) was quantified by LC/MS/MS at the Laboratory of Toxicology, Ghent Belgium; see also [76]. An appropriate method for quantification of biotin was not found for concentrations as applied here. Amino acid nitrogen data for non-analyzed time points is inferred by linearization between measured data points *t* = 3, 6, 9, 12, and 16 and the assumption that at *t* = 0 the concentration of free amino acids approaches zero, as the inoculum was only 0.5% v/v.

Trace elements were quantified by inductively coupled plasma mass spectrometry (Agilent 7700x, Santa Clara, CA) at the Trace Elements Analysis Laboratory at Dartmouth College.

### Residual medium nitrogen

Residual nitrogen in ammonia and urea was determined by the “Urea/Ammonia (Rapid) assay procedure” from Megazyme (Bray, Ireland).

## Additional files

Additional file 1: Figure S1.
**A.** Extracted ion m/z 117 trace of gas chromatography-mass spectrometry electron impact ionization (70 eV) of a microbial supernatant sample indicating the presence of A) meso-2,3-butanediol and B) RR/SS-2,3-butanediol (top panel). Their corresponding fragmentation patterns are shown in the middle and lower panels, respectively. Additional file 2: Figure S1.
**B.** Total ion current traces of gas chromatography-mass spectrometry electron impact ionization (70 eV) of standards of A) meso-2,3-butanediol and B) RR/SS-2,3-butanediol (top panel). Their corresponding fragmentation patterns are shown in the middle and lower panels, respectively. Additional file 3: Figure S2.
**A.** Elution profile (A) and electron impact (70 eV) fragmentation pattern (B) of isobutanol sample partitioned from supernatant with diethyl ether. Additional file 4: Figure S2.
**B.** Electron impact (70 eV) fragmentation pattern of isobutanol from sample versus Wiley database match. Additional file 5: Figure S2.
**C.** Elution profile (A) and electron impact (70 eV) fragmentation pattern (B) of isobutanol standard (1%) in diethyl ether solvent. Additional file 6: Figure S2.
**D.** Electron impact (70 eV) fragmentation pattern of isobutanol standard versus Wiley database.

## Abbreviations

2M1B: 2-methyl-1-butanol
3M1B: 3-methyl-1-butanol
BCA: bicinchoninic acid
CBP: consolidated bioprocessing
EI: electron ionization
MSTFA: N-Methyl-N-(trimethylsilyl)trifluoroacetamide
MTC: Medium for Thermophilic Clostridia
PABA: para-aminobenzoic acid
RI: refractive index
TMCS: trimethylchlorosilane
TMS: trimethylsilyl
