# Supplementary material for: The exometabolome of Clostridium thermocellum reveals overflow metabolism at high cellulose loading
Source: Biotechnol Biofuels. 2014 Oct 21;7:155. doi: 10.1186/s13068-014-0155-1 (PMC4207885; doi:10.1186/s13068-014-0155-1)
Supplement: Additional file 1: Figure S1. — A. Extracted ion m/z 117 trace of gas chromatography-mass spectrometry electron impact ionization (70 eV) of a microbial supernatant sample indicating the presence of A) meso-2,3-butanediol and B) RR/SS-2,3-butanediol (top panel). Their corresponding fragmentation patterns are shown in the middle and lower panels, respectively. [file 13068_2014_155_MOESM1_ESM.pdf]

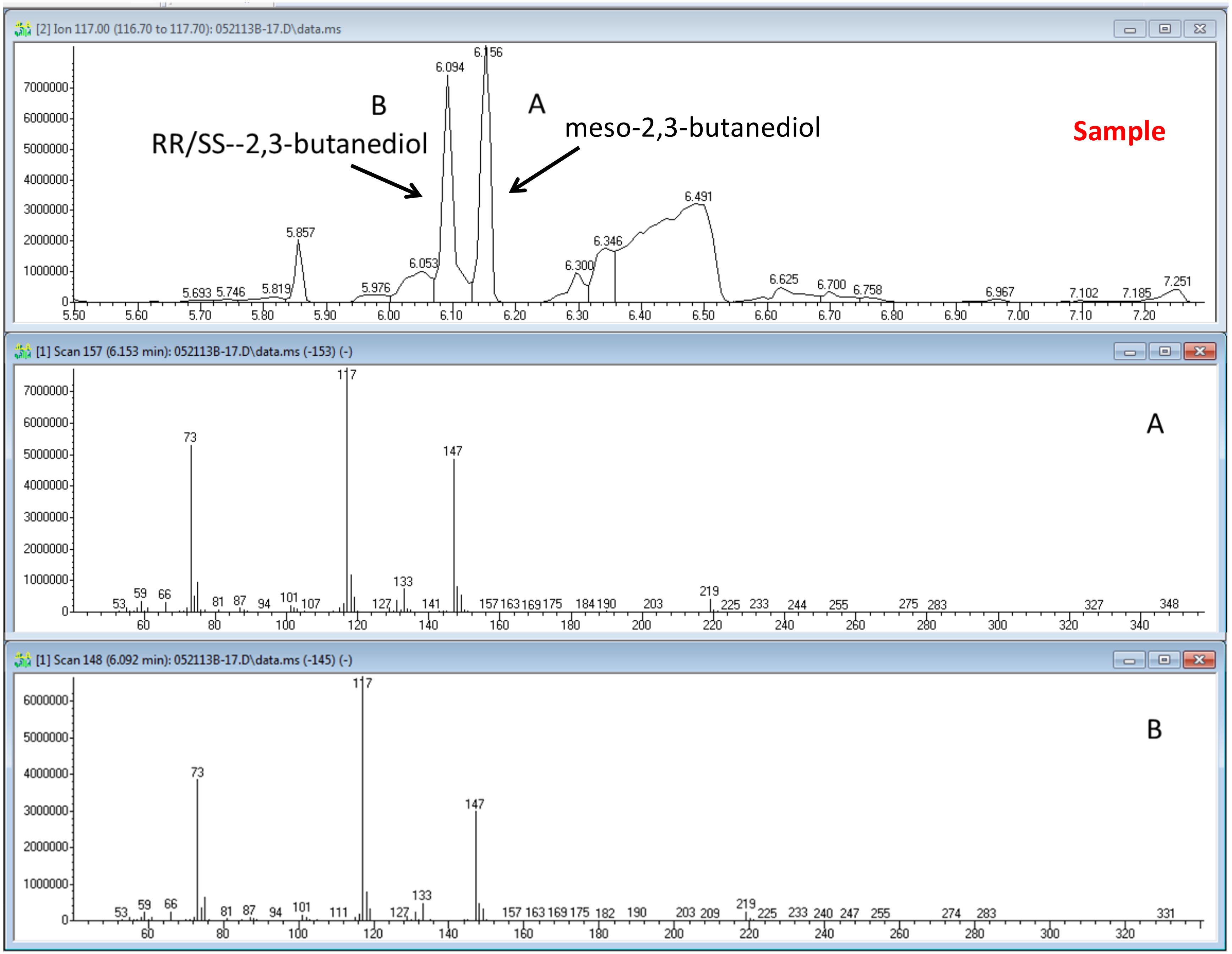

Supplemental figure 1A: *Extracted ion m/z 117 trace of gas chromatography-mass spectrometry electron impact ionization (70 eV) of a microbial supernatant sample indicating the presence of A) meso-2,3-butanediol and B) RR/SS-2,3-butanediol in the top panel, and their corresponding fragmentation patterns are shown in the middle and lower panels, respectively.*
