# Supplementary material for: The exometabolome of Clostridium thermocellum reveals overflow metabolism at high cellulose loading
Source: Biotechnol Biofuels. 2014 Oct 21;7:155. doi: 10.1186/s13068-014-0155-1 (PMC4207885; doi:10.1186/s13068-014-0155-1)
Supplement: Additional file 2: Figure S1. — B. Total ion current traces of gas chromatography-mass spectrometry electron impact ionization (70 eV) of standards of A) meso-2,3-butanediol and B) RR/SS-2,3-butanediol (top panel). Their corresponding fragmentation patterns are shown in the middle and lower panels, respectively. [file 13068_2014_155_MOESM2_ESM.pdf]

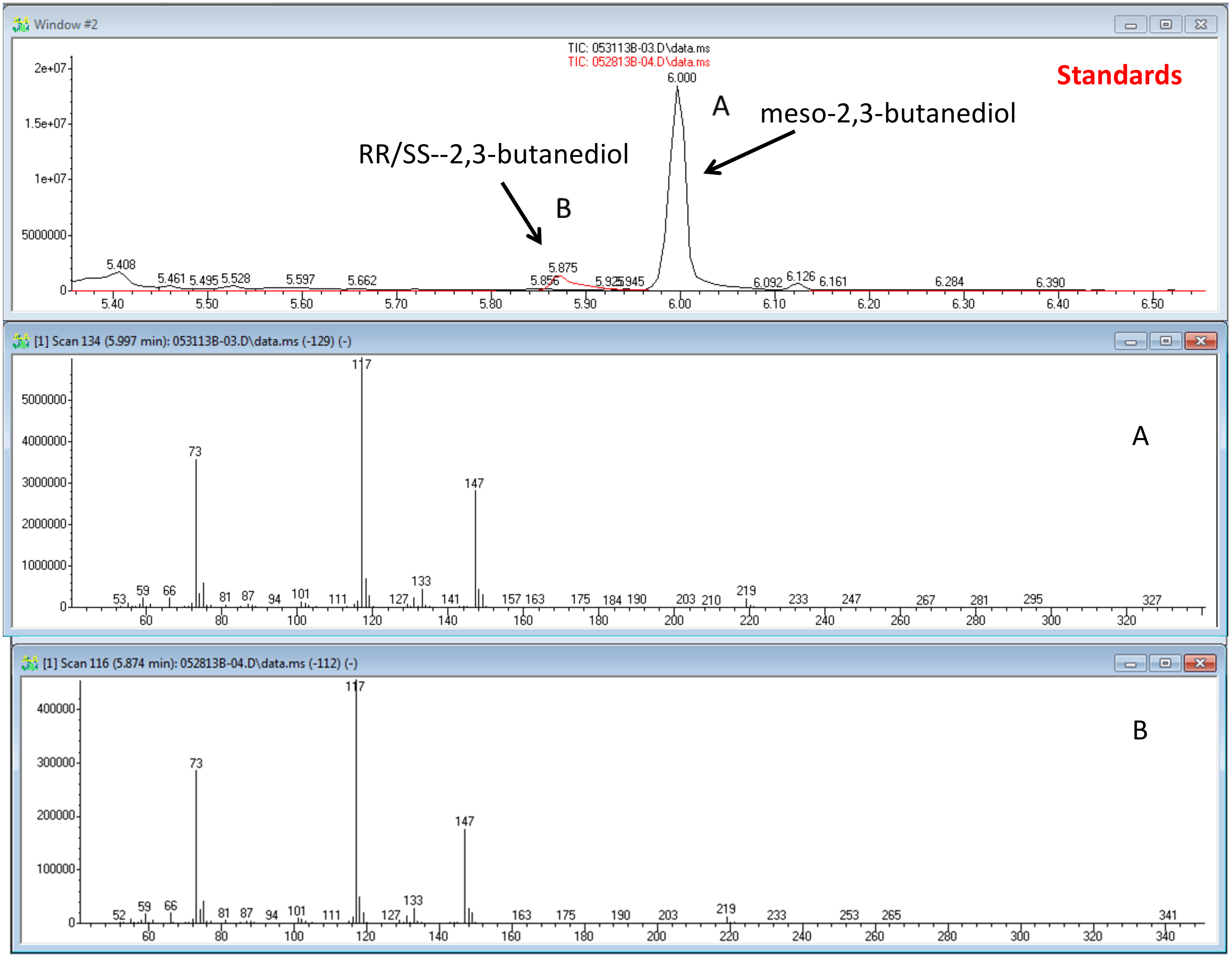

Supplemental figure 1B: *Total ion current traces of gas chromatography-mass spectrometry electron impact ionization (70 eV) of standards of A) meso-2,3-butanediol and B) RR/SS-2,3-butanediol in the top panel, and their corresponding fragmentation patterns are shown in the middle and lower panels, respectively.*
