# Supplementary material for: The exometabolome of Clostridium thermocellum reveals overflow metabolism at high cellulose loading
Source: Biotechnol Biofuels. 2014 Oct 21;7:155. doi: 10.1186/s13068-014-0155-1 (PMC4207885; doi:10.1186/s13068-014-0155-1)
Supplement: Additional file 4: Figure S2. — B. Electron impact (70 eV) fragmentation pattern of isobutanol from sample versus Wiley database match. [file 13068_2014_155_MOESM4_ESM.pdf]

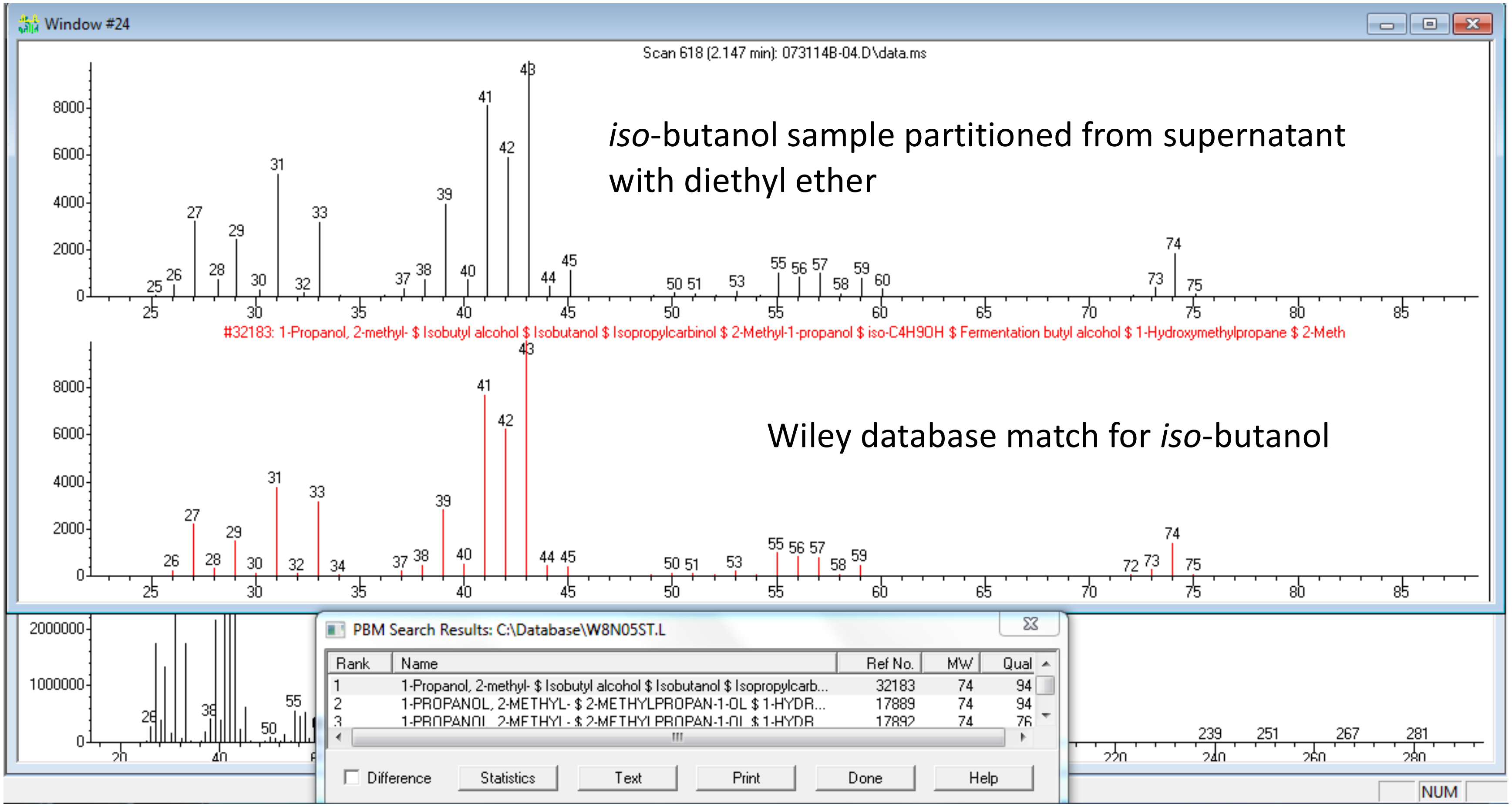

Supplemental figure 2B: *Electron impact (70 eV) fragmentation pattern of iso-butanol from sample versus Wiley database match.*
