# Supplementary material for: The exometabolome of Clostridium thermocellum reveals overflow metabolism at high cellulose loading
Source: Biotechnol Biofuels. 2014 Oct 21;7:155. doi: 10.1186/s13068-014-0155-1 (PMC4207885; doi:10.1186/s13068-014-0155-1)
Supplement: Additional file 5: Figure S2. — C. Elution profile (A) and electron impact (70 eV) fragmentation pattern (B) of isobutanol standard (1%) in diethyl ether solvent. [file 13068_2014_155_MOESM5_ESM.pdf]

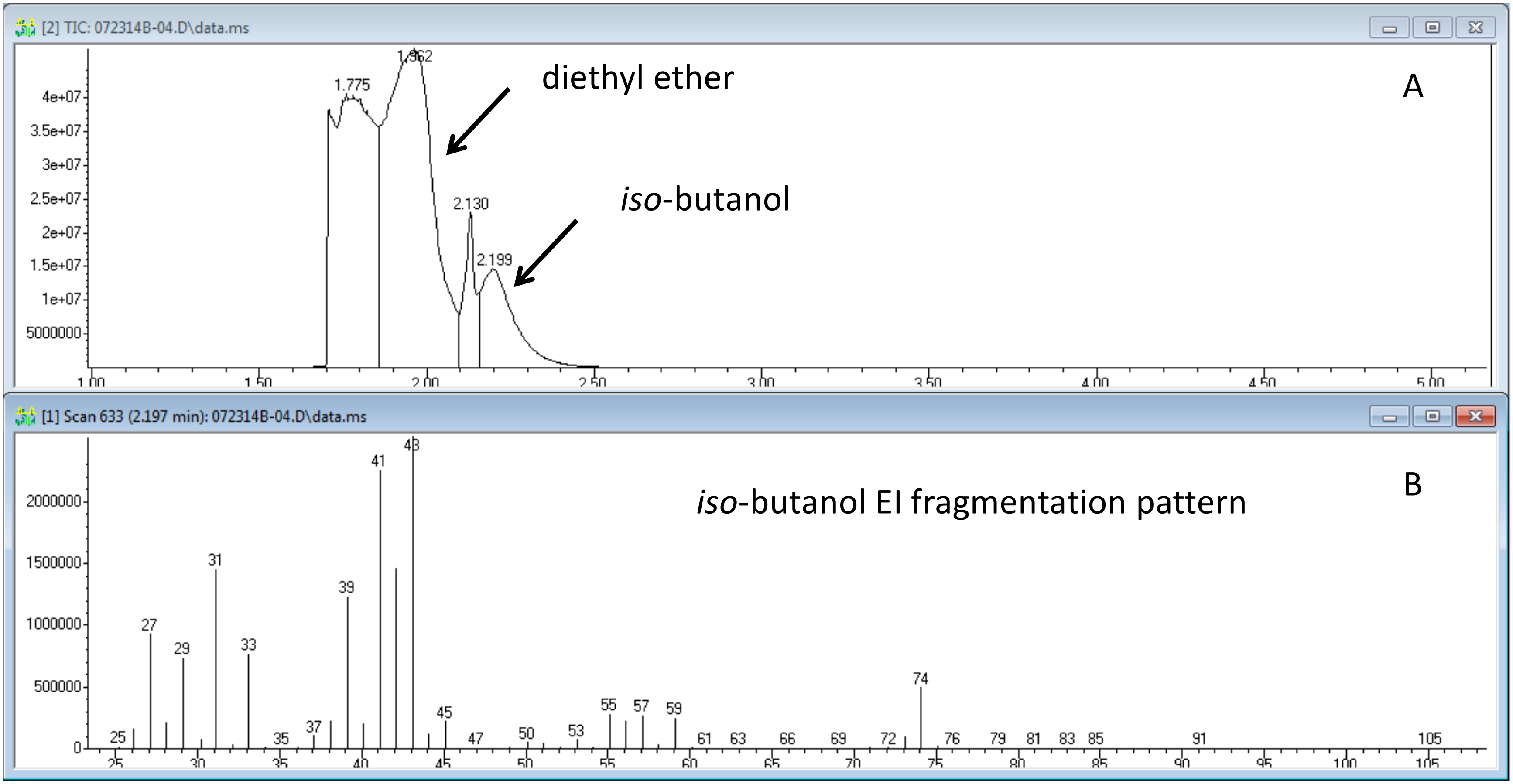

Supplemental figure 2C: *Elution profile (A) and electron impact (70 eV) fragmentation pattern (B) of iso-butanol standard (1%) in diethyl ether solvent*.
