# Supplementary material for: The exometabolome of Clostridium thermocellum reveals overflow metabolism at high cellulose loading
Source: Biotechnol Biofuels. 2014 Oct 21;7:155. doi: 10.1186/s13068-014-0155-1 (PMC4207885; doi:10.1186/s13068-014-0155-1)
Supplement: Additional file 6: Figure S2. — D. Electron impact (70 eV) fragmentation pattern of isobutanol standard versus Wiley database. [file 13068_2014_155_MOESM6_ESM.pdf]

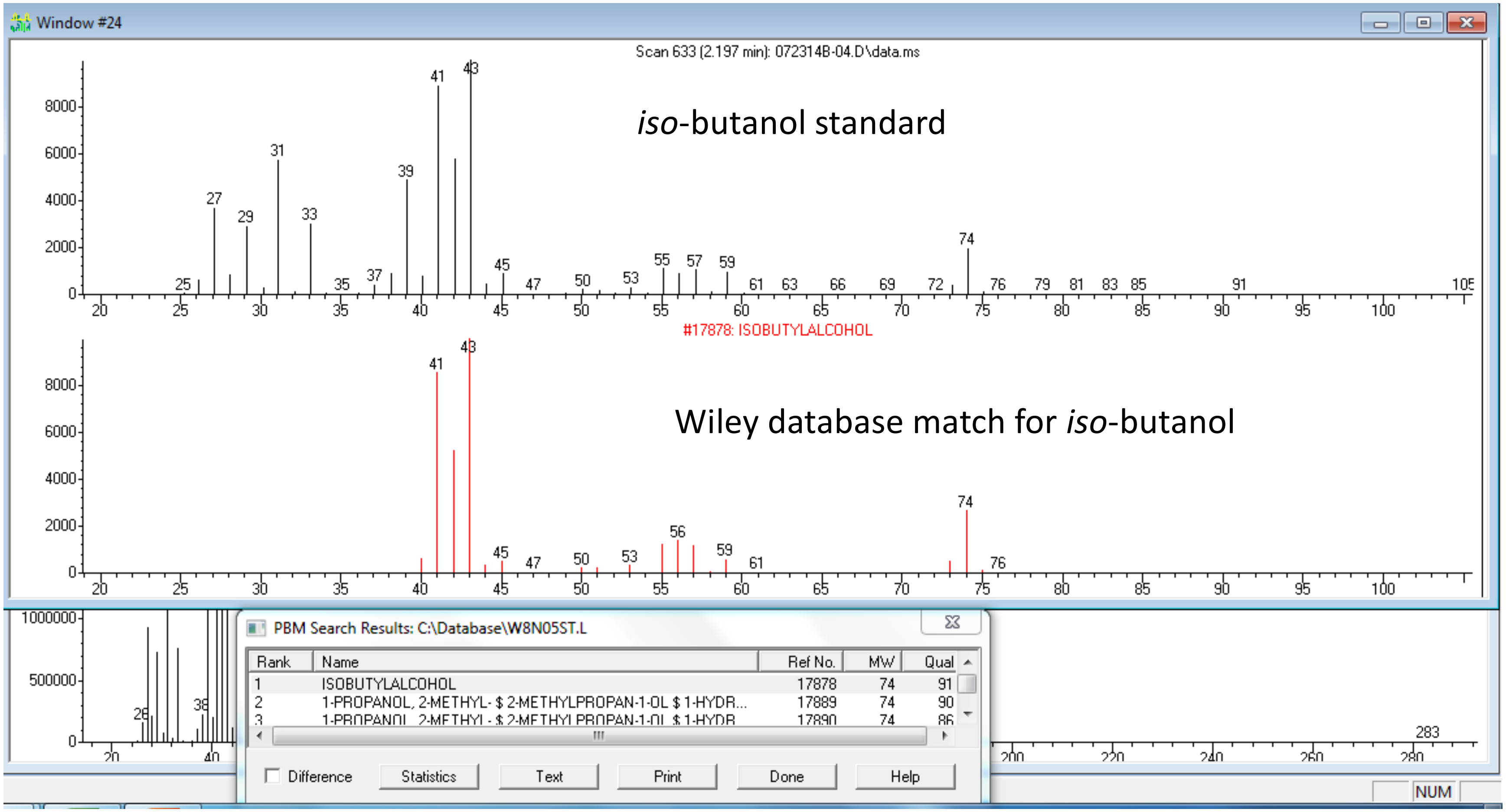

Supplemental figure 2D: *Electron impact (70 eV) fragmentation pattern iso-butanol standard versus Wiley database.*
